# Krüppel-like Factor 15 Suppresses Ferroptosis by Activating an NRF2/GPX4 Signal to Protect against Folic Acid-Induced Acute Kidney Injury

**DOI:** 10.3390/ijms241914530

**Published:** 2023-09-26

**Authors:** Xue Yang, Shihui Dong, Yun Fan, Yuanyuan Xia, Fan Yang, Zhaohong Chen, Dacheng Chen, Mingchao Zhang, Dandan Liang, Caihong Zeng

**Affiliations:** 1 National Clinical Research Center for Kidney Diseases, Jinling Hospital, Affiliated Hospital of Medical School, Nanjing University, Nanjing 210044, China; 2 Jinling Clinical Medical College, Nanjing Medical University, Nanjing 210008, China

**Keywords:** acute kidney injury, KLF15, NRF2, ferroptosis, GPX4

## Abstract

Acute kidney injury (AKI) is a common and serious disease with high morbidity and mortality, and its pathophysiological mechanisms are not fully understood. Increasing evidence suggests an important role of ferroptosis in AKI. Krüppel-like factor 15 (KLF15) is a transcription factor involved in several metabolic diseases, but its role in AKI and ferroptosis remains unclear. In this study, we explored the potential role of KLF15 using a folic acid-induced AKI model. Our study showed that KLF15 expression was reduced in kidney tissues of AKI mice, and KLF15 knockout exacerbated folic acid-induced ferroptosis and kidney injury. In vitro studies revealed that the ferroptosis inducer erastin significantly suppressed KLF15 expression in human tubular epithelial cells. Notably, the overexpression of KLF15 attenuated ferroptosis, as evidenced by a decrease in the lipid peroxidation marker of malondialdehyde and the upregulation of glutathione peroxidase 4 (GPX4), while KLF15 knockdown with shRNA exerted the opposite effect. Mechanistically, KLF15 stabilized the protein of nuclear factor erythroid 2-related factor 2 (NRF2) and subsequently increased the GPX4 level. Collectively, KLF15 plays an important role in the modulation of ferroptosis in AKI and may be a potential therapeutic target for treating AKI.

## 1. Introduction

Krüppel-like factor 15(KLF15), also known as kidney Krüppel-like factor (KKLF), is enriched in the kidney and is a member of the zinc finger family of transcription factors. Similar to other KLF family members, KLF15 contributes to cell proliferation, apoptosis, and mitochondrial biogenesis. KLF15 is involved in the regulation of various metabolic pathways, including lipid metabolism [1]. In cardiomyocytes and skeletal muscle, KLF15 knockout reduced palmitate utilization due to the dysregulation of several fatty acid β-oxidation (FAO)genes, and KLF15 was shown to interact with PPARα or PPARδ to actively transcribe FAO genes [2,3,4]. Recent studies have demonstrated that the knockdown of KLF15 worsens ferroptosis, oxidative stress, and lipid peroxidation by suppressing the NRF2/SLC7A11/GPX4 signaling pathway [5,6]. Consequently, KLF15 is implicated in both normal renal physiology and pathological development, as evidenced by a growing body of research [7,8,9].

Aristolochic acid I treatment significantly reduces KLF15 expression in proximal tubule cells [10,11]. Compared to control mice, the proximal tubule-specific deletion of KLF15 enhanced proximal tubule injury and renal function decline due to the loss of interaction with PPARα, leading to the loss of FAO gene transcription [10]. Furthermore, KLF15 directly binds enhancers and promotes the transcription of regenerative genes, such as adrenoreceptor alpha 1A. Conversely, inhibiting KLF15 activity impedes nephric tubule regeneration [12]. Despite these findings, the precise mechanisms underlying the protective effects of KLF15 on proximal tubule injury remain largely unclear.

Ferroptosis, a new typical form of regulated cell death, is characterized by the excessive accumulation of lipid peroxidation in an iron-dependent manner [13]. There are three main regulatory mechanisms of ferroptosis [14,15,16]: lipid peroxidation, the consumption of glutathione peroxidase 4 (GPX4) and a cystine/glutamate antiporter system, and iron overload. The pathophysiology of ferroptosis is multifaceted, and recent studies have shown that several transcription factors are related, including nuclear factor erythroid 2-related factor 2 (NRF2) [17,18,19]. Notably, NRF2 restores redox homeostasis by activating several antioxidant response element (ARE)-containing genes, including GPX4, which is an essential antiferroptotic transcriptional regulator that limits the generation of lipid peroxides [20]. Glutathione (GSH) fuels the catalytic consumption of GPX4. The cysteine required for the formation of GSH is provided by the amino acid transporter SLC7A11. Thus, GPX4 and SLC7A11 are regarded as important inhibitors of ferroptosis. It has been demonstrated that ferroptosis can be triggered in a variety of renal diseases including acute kidney injury (AKI), renal clear cell carcinoma, and diabetic nephropathy [21,22]. However, the underlying mechanisms involved in ferroptosis and AKI have not been elucidated.

In regard to the essential role of KLF15 in regulating lipid metabolism and lipid disturbance in ferroptosis, as stated above, we hypothesized that KLF15 could be associated with the development of AKI and ferroptosis. In this study, we aimed to investigate whether KLF15 was involved in ferroptosis-related tubular injury. The expression of KLF15 was detected in human tubular epithelial cells following erastin treatment. In addition, ferroptosis-related events were detected when KLF15 was silenced or overexpressed. We also generated KLF15-KO mice to validate our hypothesis in vivo. Our results may contribute to the identification of novel therapeutic targets for AKI.

## 2. Results

### 2.1. KLF15 Expression Was Decreased in AKI Mice and KLF15 Knockout Aggravated Kidney Injury In Vivo

**Figure 1:**
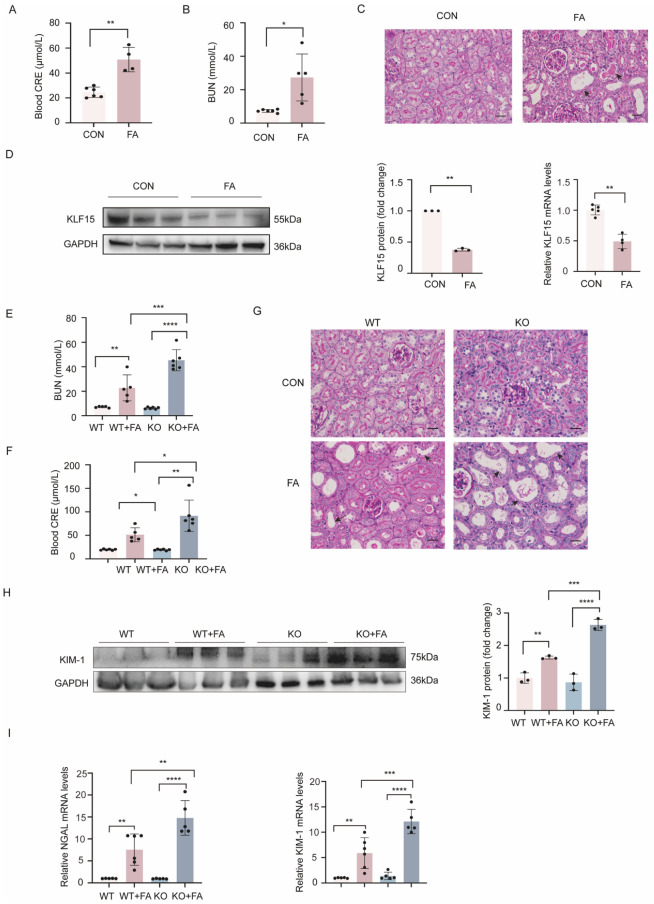
KLF15 expression is reduced in AKI mice, and KLF15 deficiency promotes renal injury in mice. (**A**,**B**) Serum CRE and BUN levels in each group. (**C**) Periodic acid-Schiff (PAS) staining showed a significant acute tubular injury in the FA kidney. (**D**) Protein and mRNA expression of KLF15 in kidney samples of FA mice. (**E**,**F**) Serum BUN and CRE levels in each group. (**G**) Representative images of PAS staining. (**H**) Protein expression of KIM-1 in kidney samples in each group. (**I**) KIM-1 and NGAL mRNA expression in kidney samples in each group. KLF15, Krüppel-like factor 15; CRE, creatinine; BUN, blood urea nitrogen; CON, control; FA, folic acid; KIM-1, kidney injury molecule 1; NGAL, neutrophil gelatinase-associated lipocalin. Data are shown as the mean ± SD (*n* = 5). Original magnification, ×400 for all. Scale bar = 50 μm. * *p* < 0.05, ** *p* < 0.01, *** *p* < 0.001, **** *p* < 0.0001.

There is growing evidence that ferroptosis is important in the development of AKI [23,24]. Folic acid injections were used to establish an AKI model in C57BL/6 wild-type mice. FA treatment induced acute kidney injury in wild-type mice, as evidenced by significant increases in serum blood urea nitrogen (BUN) and creatinine levels and tubular injury (Figure 1A–C). Furthermore, there was a significant fold-change both in the expression of KLF15 protein and in mRNA in FA-treated mice (Figure 1D). Next, the role of KLF15 in AKI was examined using KLF15-knockout mice in the FA-AKI model. Apparently, KLF15 deficiency aggravated the loss of renal function and tubular damage induced by folic acid. In comparison to wild type mice treated with folic acid, we observed that the KLF15-KO group exhibited significant increases in serum BUN and creatinine levels and tubular damage (Figure 1E–G). Consistent with these data, Western blotting showed that KLF15 deficiency could promote the FA-induced upregulation of the KIM-1 protein expression (Figure 1H). Moreover, KLF15 deficiency promoted an increase in the mRNA levels of cortical KIM-1 and neutrophil gelatinase-associated lipocalin (NGAL), which correlated with the kidney damage (Figure 1I).

### 2.2. KLF15 Knockout Aggravated FA-Induced Ferroptosis In Vivo

**Figure 2:**
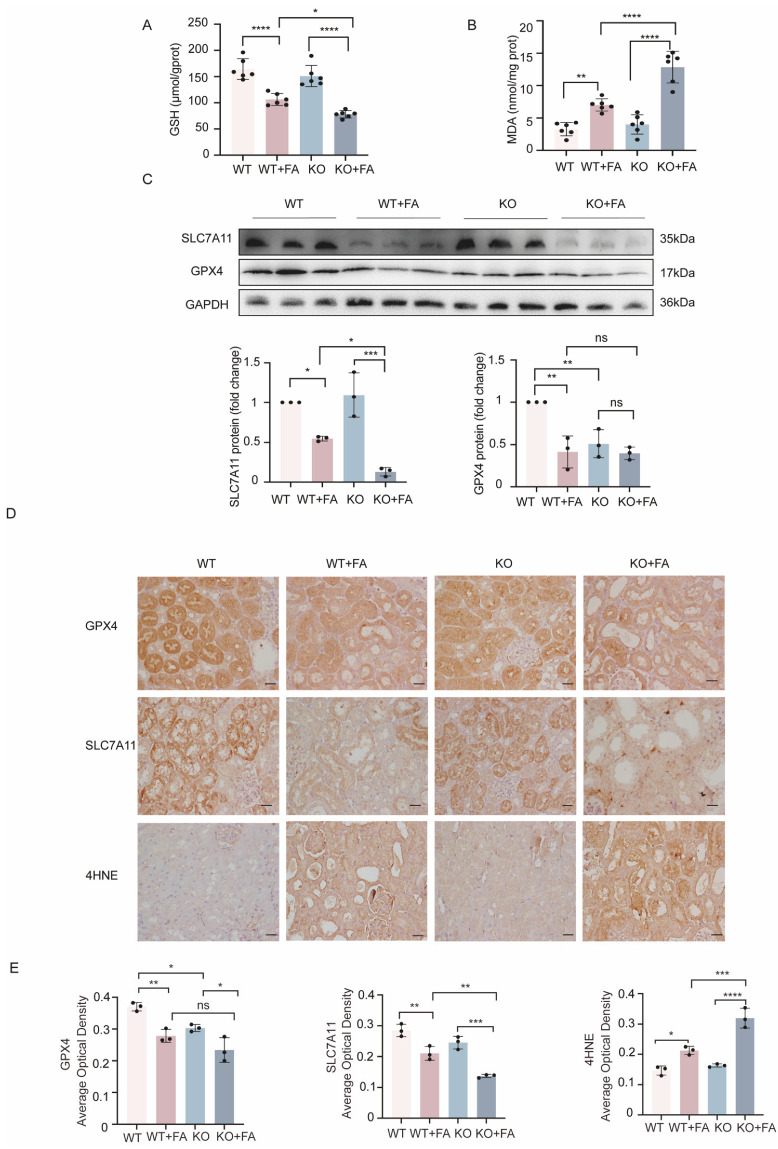
KLF15 deficiency promotes ferroptotic responses in mice. (**A**,**B**) GSH and MDA levels in kidney tissues. (**C**) Renal protein levels of GPX4 and SLC7A11 were detected by Western blotting. (**D**) Representative immunohistochemistry images showing the expression of GPX4, SLC7A11, and 4HNE. (**E**) The quantification of the positive cells of specific signals. The data are shown as the mean ± SD (*n* = 6). KLF15, Krüppel-like factor 15; CON, control; FA, folic acid; GSH, glutathione; MDA, malonaldehyde; GPX4, glutathione peroxidase 4; 4HNE, 4-hydroxynonenal. One-way ANOVA and Bonferroni post hoc tests were used for statistical analysis. Original magnification, ×400 for all. Scale bar = 50 μm.* *p* < 0.05, ** *p* < 0.01, *** *p* < 0.001, **** *p* < 0.0001.

Lipid peroxidation levels were assessed to determine the importance of ferroptosis in FA-AKI. As expected, GSH levels were decreased in KLF15-KO mice given folic acid injections, whereas MDA levels showed the opposite effect (Figure 2A,B). The protein levels of SLC7A11 and GPX4 were reduced in wild type mice, and SLC7A11 protein expression was nearly absent in the KLF15-KO group after FA treatment. However, GPX4 protein levels were unchanged by KLF15 deficiency (Figure 2C). IHC staining revealed considerably reduced levels of SLC7A11 and GPX4 expression, while lipid peroxidation, as assessed by 4-hydroxynonenal (4HNE), was significantly higher in the kidneys of FA animals than in those of control mice. The effects on SLC7A11 and GPX4 expression were all markedly exacerbated in the KLF15-KO group following FA-treatment. When exposed to FA toxicity, 4HNE levels in kidney tissue were similarly significantly higher in KLF15-knockout mice than in wild type mice (Figure 2D,E). Most notably, we observed that KLF15 deficiency reduced GPX4 expression in the renal tissue of KLF15-KO mice in the absence of folic acid injection. Taken together, our results indicate that KLF15 knockout promotes ferroptosis and increases FA-induced kidney injury in vivo.

### 2.3. KLF15 Is Reduced in Erastin-Treated HK2 Cells

**Figure 3:**
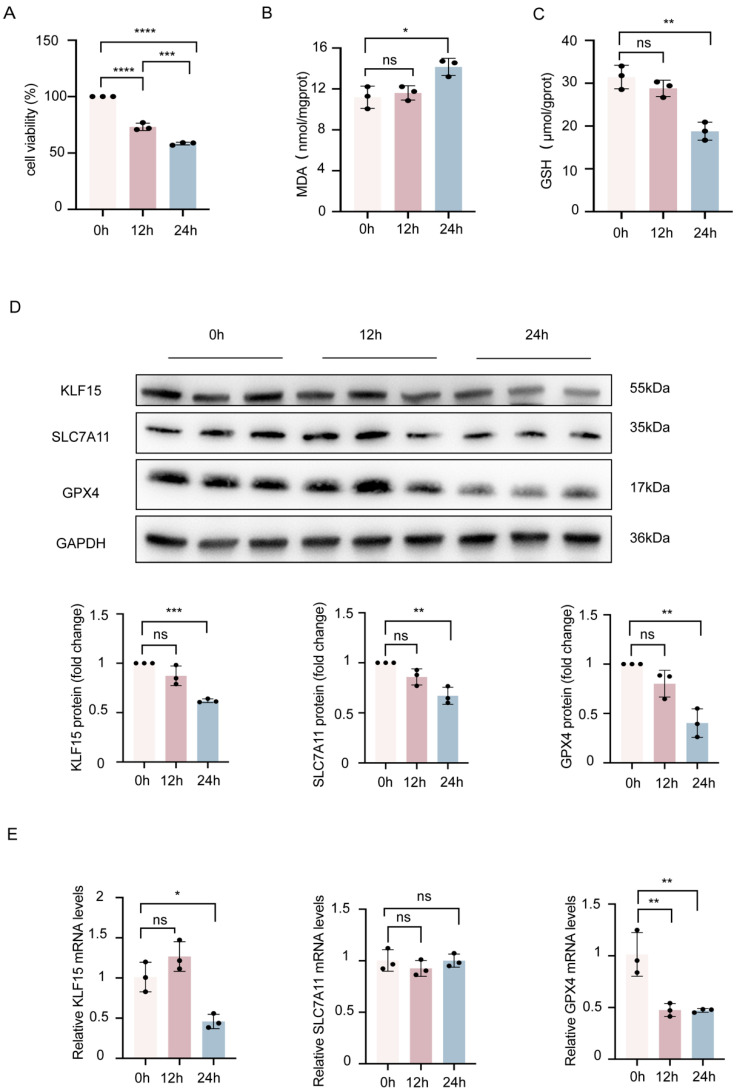
KLF15 is reduced in erastin-treated HK2 cells. (**A**) HK2 cells subjected to erastin (1 μmol/L) were cultured for 12 and 24 h, and cell viability was measured. (**B**,**C**). Levels of MDA and GSH in each group at 12 h or 24 h after erastin treatment. (**D**) The expression of KLF15, GPX4, and SLC7A11 in the indicated cells was tested by Western blotting. GAPDH was used as a loading control. (**E**) Relative mRNA expression of KLF15, SLC7A11, and GPX4 detected by RT-qPCR in HK2 cells treated with erastin for 12/24 h. KLF15, Krüppel-like factor 15; HK2, human tubular epithelial cells; GSH, glutathione; MDA, malonaldehyde; GPX4, glutathione peroxidase 4. Values are expressed as the mean ± SD. *n* = 3, * *p* < 0.05, ** *p* < 0.01, *** *p* < 0.001, **** *p* < 0.0001, compared to control.

To investigate the features of KLF15 expression during ferroptosis, 1 μmol/L erastin was used to induce ferroptosis. We first treated HK2 cells with erastin for different times and found that treatment with erastin for 24 h significantly decreased the viability of HK2 cells in comparison to cells cultured under normal conditions (Figure 3A). We then identified the lipid-associated products and reactive oxygen species (ROS) in the cells because lipid peroxidation is characteristic of ferroptosis. Malondialdehyde (MDA) and GSH preserve the balance between oxidation and reduction. After 24 h, the erastin-induced increase in MDA was accompanied by a reduction in GSH in HK2 cells (Figure 3B,C).

We then measured the expression of KLF15 and the ferroptosis markers SLC7A11 and GPX4 and found that the relative protein expression of KLF15, SLC7A11, and GPX4 protein levels was significantly reduced in HK2 cells exposed to erastin for 24 h (Figure 3D). Consistent with the protein expression results, RT-qPCR showed a significant decrease in KLF15 and GPX4 mRNA expression (Figure 3E).

### 2.4. KLF15 Protects HK2 Cells from Erastin-Induced Ferroptosis

**Figure 4:**
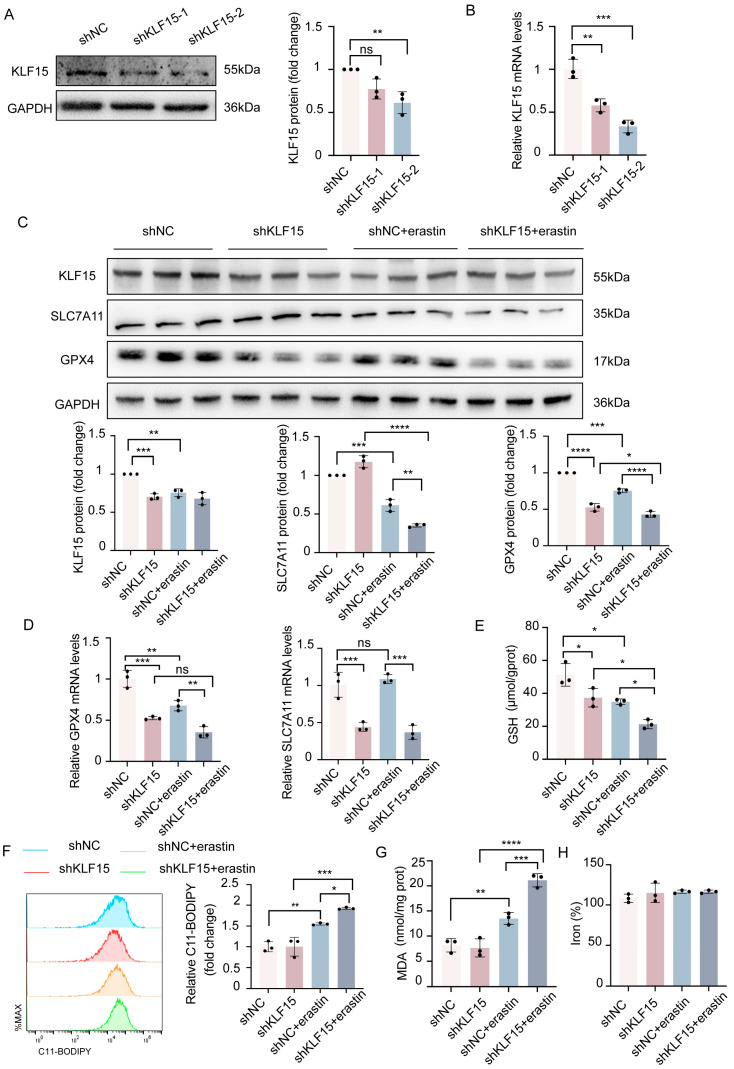
Silencing of KLF15 enhances ferroptosis in HK2 cells. HK2 cells were transfected with KLF15 shRNA or the control vector and then treated with erastin (1 μmol/L, 24 h). (**A**,**B**) KLF15 protein and mRNA expression was detected in HK2 cells. (**C**,**D**) The protein and mRNA expression of SLC7A11 and GPX4 in each group. (**E**–**H**) Ferroptosis-associated markers, GSH, lipid ROS, MDA, and iron levels were assayed. KLF15, Krüppel-like factor 15; HK2, human tubular epithelial cells; GSH, glutathione; MDA, malonaldehyde; GPX4, glutathione peroxidase 4. Values are expressed as the means ± SD. *n* = 3, * *p* < 0.05, ** *p* < 0.01, *** *p* < 0.001, **** *p* < 0.0001.

Although previous studies have demonstrated that KLF15 plays a protective role in renal tubular cells, its function in ferroptosis remains unclear. We initially generated two distinct short hairpin RNAs (shRNAs) targeting KLF15 (shKLF15-1 and shKLF15-2), evaluated their knockdown efficiency in HK2 cells, and attempted to determine the function of KLF15 in HK2 cells. RT-qPCR and Western blotting results showed effective shRNA-mediated KLF15 gene knockdown in HK2 cells, particularly by shKLF15–2 (Figure 4A,B). The expression of KLF15 was silenced in HK2 cells, which were then treated with erastin (1 μmol/L, 24 h) to trigger ferroptosis. Western blot analysis was used to evaluate the levels of GPX4 and SLC7A11 (Figure 4C). We observed that KLF15 knockdown exacerbated the decreased expression of SLC7A11 and GPX4 induced by erastin. Consistent with the protein levels, silencing KLF15 decreased in GPX4 and SLC7A11 mRNA expression in treated HK2 cells (Figure 4D). In addition, the levels of GSH depletion and MDA and iron accumulation were also measured (Figure 4E,G,H). The results showed that the GSH levels in KLF15 shRNA-transfected cells were considerably lower than those in NC shRNA-transfected cells (Figure 4E). Similarly, the levels of MDA in HK2 cells transfected with KLF15 shRNA were significantly higher than those in NC shRNA-transfected cells treated with erastin (Figure 4G). However, no obvious differences in intracellular iron were found in the groups (Figure 4H). Excessive oxidative stress that results in lipid peroxidation and a breakdown of membrane permeability is what defines ferroptosis. Lipid ROS was used to evaluate this condition. The results demonstrated that the treatment of HK2 cells with erastin increased lipid ROS, and suppressing KLF15 could enhance this increase (Figure 4F).

**Figure 5:**
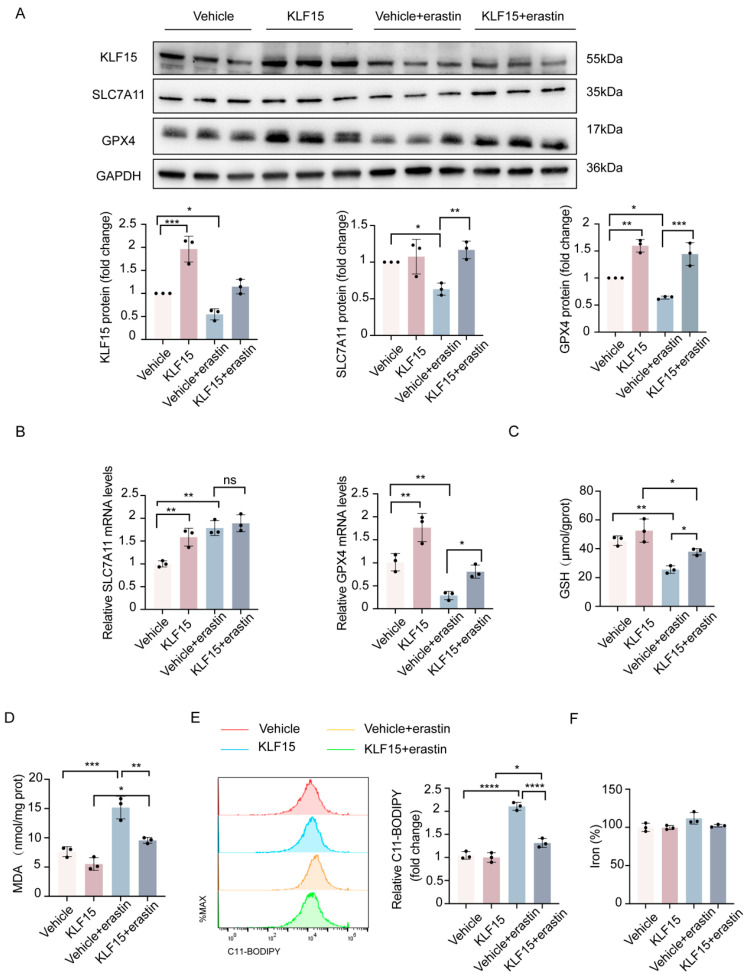
KLF15 overexpression ameliorates ferroptosis in HK2 cells. (**A**,**B**) Protein expression and mRNA levels of KLF15, SLC7A11, and GPX4 in each group. (**C**–**F**) Ferroptosis-associated markers, GSH, MDA, lipid ROS, and iron levels were assessed. KLF15, Krüppel-like factor 15; HK2, human tubular epithelial cells; GSH, glutathione; MDA, malonaldehyde; GPX4, glutathione peroxidase 4. Values are expressed as the means ± SD. *n* = 3, * *p* < 0.05, ** *p* < 0.01, *** *p* < 0.001, **** *p* < 0.0001.

To further determine whether KLF15 is implicated in the ferroptosis of renal tubular cells, we investigated the impact of KLF15 overexpression on renal tubular cell ferroptosis induced by erastin. RT-qPCR analysis and Western blotting were performed on the erastin-treated HK2 cells in the presence or absence of KLF15 overexpression. We observed that the protein expression of SLC7A11 and GPX4 protein was significantly ameliorated when KLF15 was overexpressed (Figure 5A). However, the induction of KLF15 significantly ameliorated the decrease in the mRNA expression of GPX4 but not SLC7A11 (Figure 5B). In addition, the decrease in GSH levels induced by erastin was alleviated by transient KLF15 overexpression (Figure 5C). Lipid peroxidation was also attenuated in KLF15-overexpressing HK2 cells (Figure 5D,E), while there were no overt variations in the intracellular iron levels in any of the groups (Figure 5F). These results collectively suggest that KLF15 protects against erastin-induced ferroptosis in HK2 cells.

### 2.5. KLF15 Inhibits Ferroptosis by Activating the NRF2/GPX4 Signaling Axis

**Figure 6:**
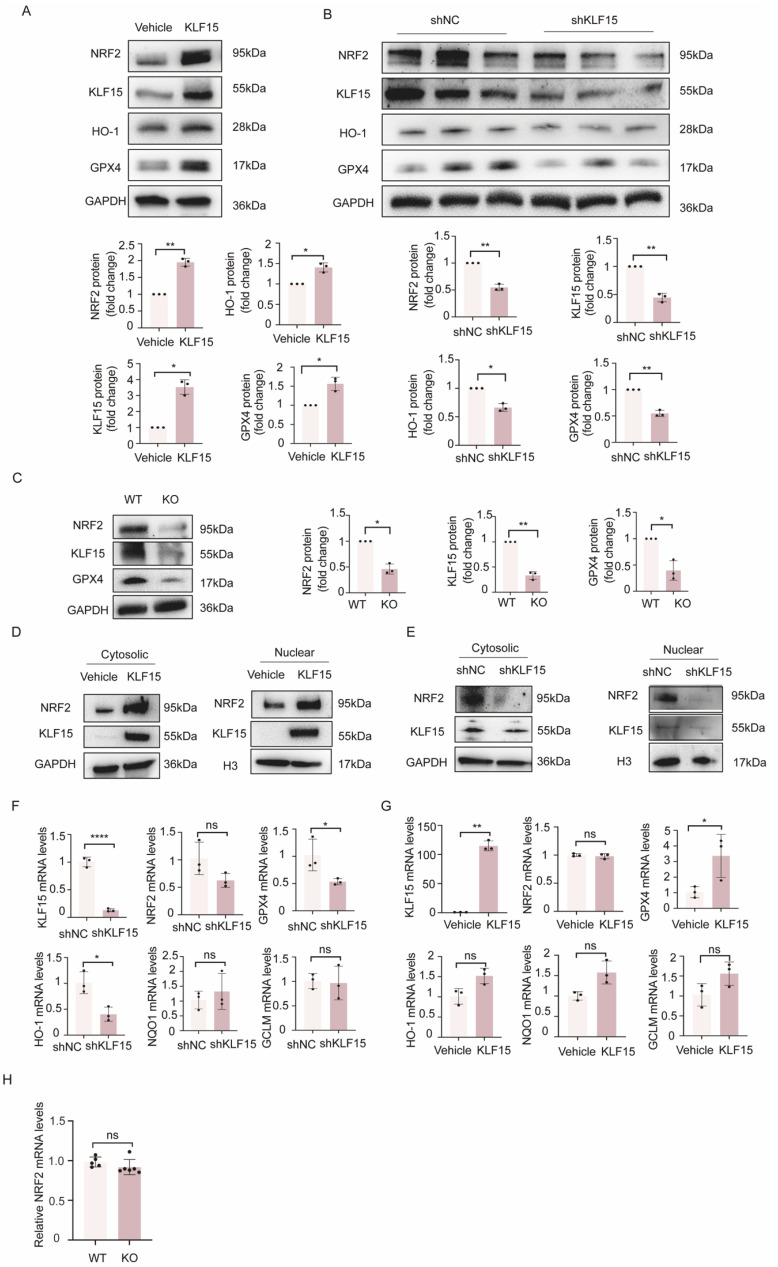
KLF15 post-transcriptionally activates NRF2. (**A**,**B**) NRF2 expression was analyzed in KLF15-overexpressing or KLF15 knockdown HK2 cells by Western blotting. (**C**). NRF2 and GPX4 protein expression was measured in kidney samples of KLF15-knockout mice. (**D**,**E**) Western blot analysis of NRF2 in the cytosolic and nuclear fractions of KLF15-overexpressing or KLF15 knockdown in HK2 cells. GAPDH and histone H3 were used as loading controls. (**F**,**G**) RT-qPCR was used to detect the expression of NRF2 and its target genes, including GPX4, HO-1, NQO1, and GCLM. (**H**) NRF2 mRNA expression was measured in kidney samples of KLF15-knockout mice. KLF15, Krüppel-like factor 15; NRF2, nuclear factor erythroid 2-related factor 2; GPX4, glutathione peroxidase 4; HO-1, heme oxygenase-1; NQO1, NADPH quinone oxidoreductase 1; GCLM, glutamate-cysteine ligase modifier subunit. * *p* < 0.05, ** *p* < 0.01, **** *p* < 0.0001.

Based on the findings that KLF15 activation may prevent ferroptosis by influencing GPX4, it was reported that the transcription factor NRF2 encourages the transcription of GPX4 to inhibit ferroptosis [25]. There is further evidence that KLF2 inhibits the oxidative response by activating NRF2 [26]. It is conceivable that KLF15 also exerts protective effects on ferroptosis via the activation of NRF2.We then investigated how KLF15 overexpression or knockdown affected the expression of NRF2 and its downstream proteins in HK2 cells. As shown in Figure 6A,B, KLF15 depletion drastically reduced the protein expression of NRF2, GPX4, and heme oxygenase-1 (HO-1). Conversely, the overexpression of KLF15 significantly elevated the protein levels of NRF2, GPX4, and HO-1. We found that NRF2 protein levels were substantially decreased in the kidney tissue of KLF15-KO mice compared to those of normal controls (Figure 6C), which is consistent with the Western blot results in vitro.

How KLF15 affects NRF2 activity was examined, and further studies revealed that the overexpression of KLF15 in HK2 cells clearly increased the cytosolic and nuclear protein levels of NRF2 (Figure 6D). Conversely, NRF2 protein levels in the cytosol and nucleus were reduced when KLF15 was knocked down (Figure 6E). RT-qPCR analysis showed that the knockdown or overexpression of KLF15 did not affect NRF2 mRNA levels. There was a significant change in GPX4 mRNA, while no changes were observed in the mRNA levels of NQO1 and GCLM (Figure 6F,G). The level of NRF2 mRNA in the kidney tissue of KLF15 knockout mice also did not change significantly (Figure 6H). These findings suggest that the renal protective effects of KLF15 may involve the activation of the NRF2/GPX4 signaling pathway and that KLF15 does not affect NRF2 genetically.

### 2.6. KLF15 Stabilizes NRF2 by Suppressing Ubiquitin-Mediated Degradation

Our findings indicated that KLF15 could activate NRF2 post-transcriptionally. NRF2 can be degraded through the proteasome system by interacting with Kelch-like ECH-associated protein 1 (KEAP1), which can form an E3 ubiquitin ligase complex with cullin-3 (Cul3) [27]. Western blotting showed that the effect of KLF15 overexpression or knockdown did not affect the protein expression of KEAP1 (Figure S1). Then, we hypothesized that KLF15 could interact with NRF2 to sterically obstruct NRF2 and KEAP1 interactions. However, we performed the immunoprecipitation and found that there was no evidence of a protein-protein interaction between KLF15 and NRF2 in vitro. (Figure S2).

**Figure 7:**
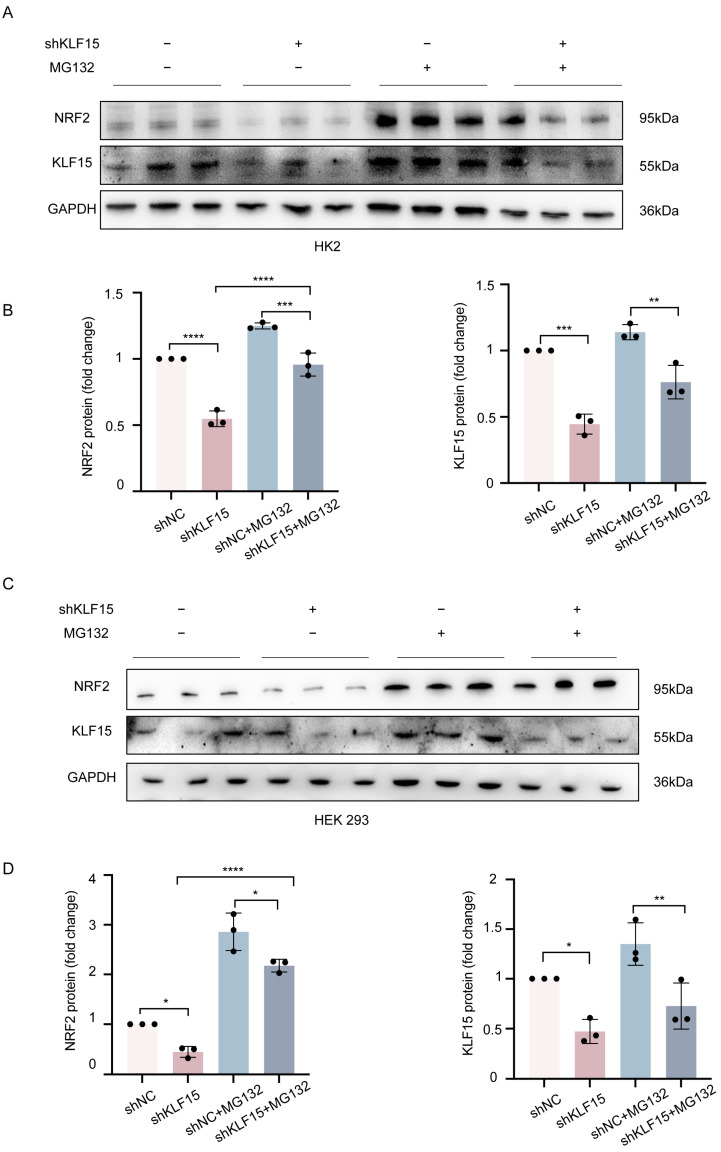
KLF15 attenuates the ubiquitin-mediated degradation of NRF2. (**A**–**D**) KLF15 knockdown HK2 and HEK 293 cells and control cells were treated with 10 μM of MG132 or DMSO for 2 h, and Western blot analysis was then performed to assess the protein expression of KLF15, NRF2, and GAPDH (loading control). KLF15, Krüppel-like factor 15; NRF2, nuclear factor erythroid 2-related factor 2; HK2, human tubular epithelial cells. * *p* < 0.05, ** *p* < 0.01, *** *p* < 0.001, **** *p* < 0.0001.

To determine the precise mechanism, we blocked the proteolytic activity of the 26S proteasome complex by treating HK2 and HEK 293 cells with MG132 to assess the effect of KLF15 on NRF2 protein stability. According to our findings, MG132 obviously reversed the inhibitory effect of KLF15 knockdown on NRF2 expression compared to the control (Figure 7A–D), Thus, it is possible that the KLF15-mediated activation of NRF2 is indirectly enhanced through other mechanisms instead of influencing KEAP1 expression or interacting with each other in kidney cells. Overall, these findings indicate that KLF15 can stabilize NRF2 by attenuating its ubiquitin-mediated degradation.

## 3. Discussion

AKI is defined as a transient decline in renal function that results in a severe clinical syndrome. AKI is especially common in hospitalized patients and is generally recognized as a potential risk factor for chronic kidney disease, cardiovascular disease, and end-stage renal disease [28,29,30]. Given its high morbidity and mortality, as well as the high burden of complications, the identification of novel targets or drugs for AKI is desperately needed. Folic acid-induced kidney disease is a classical mouse model of AKI, it has been described in humans, and the experimental model replicates almost all of the major processes in human AKI [31]. In 2017, Martin-Sanchez et al. [32] reported that ferroptosis contributed to the occurrence of folic acid-induced AKI. Previous studies have demonstrated that inhibiting ferroptosis by modulating the expression of the circadian clock components Rev-erb-α/β and repurposing antioxidants as antiferroptotic agents exerts renal protective effects in experimental models of folic acid-induced AKI [33,34,35]. Consistent with the previous research, our data showed that FA treatment led to conceivable changes in ferroptosis-related markers, and a decrease in KLF15 protein levels was observed in kidneys in the FA group.

Notably, KLF15 is a multifunctional transcription cofactor associated with various bioprocesses, and recent publications have revealed that it is involved in the modulation of lipid flux and systemic metabolic homeostasis [36,37,38]. KLF15 is known to be downregulated in multiple established kidney injury models [9,10,12,39]. Numerous studies have confirmed that KLF15 has a protective effect against kidney diseases. The majority of those studies largely focused on podocyte injury diseases or renal fibrosis. For instance, KLF15 deficiency in Foxd1-positive stromal cells was detrimental through the activation of the Wnt/β-catenin signaling pathway [40]. Quite a few studies have investigated the role of KLF15 in clinical AKI or in vivo models. In 2021, Piret et al. [10] proposed that KLF15 mediated the inflammatory response in kidney ischemia-reperfusion injury for the first time. However, the correlation between KLF15 and ferroptosis regulation has not been fully explained. In this study, folic acid-induced ferroptotic phenotypes and kidney injuries were exacerbated by KLF15 gene disruption. Further investigation showed that KLF15 overexpression reduced the lipid ROS levels and ameliorated the loss of GPX4 expression in HK2 cells. Other ferroptosis characteristics, including GSH depletion and MDA expression, also demonstrated that the upregulation of KLF15 could reduce ferroptosis levels. The lack of change in iron accumulation is consistent with a previous report [35]. Taken together, these data suggest that KLF15 plays a positive regulatory role in FA-induced AKI by inhibiting ferroptosis.

Emerging studies have revealed that GPX4 is one of the major ferroptosis defense systems [41,42,43]. Interestingly, we observed a relationship between KLF15 and GPX4. It should be noted that the expression of GPX4 was considerably reduced in the renal tissue of KLF15-KO mice without folic acid injection, which was consistent with the data at the cellular level. Many studies have shown that the deletion of GPX4 can greatly increase the sensitivity of cells to ferroptosis [42,43,44]. Angeli et al. [42] found that GPX4 disruption could cause acute renal failure and ferroptotic cell death for the first time. However, no obvious renal pathological damages or signs of ferroptosis were observed in KLF15 knockout mice. The related mechanisms behind this observation are not completely known, and they need to be further explored. NRF2, which is an antioxidant transcription factor, plays a crucial role in mediating the ferroptotic response [45]. Many components of the ferroptosis cascade are target genes of NRF2, including GPX4 [25]. To address this, we examined whether KLF15 could regulate GPX4 expression via NRF2 signaling. Previous studies have extensively demonstrated that the overexpression of KLF2 effectively activates NRF2/ARE signaling, promoting the transcription of NRF2 target genes and thus suppressing oxidative stress [26]. As expected, KLF15 overexpression also increased the expression of NRF2 and its target gene HO-1. These results demonstrated that KLF15 expectedly enhanced NRF2 protein stability, as expected. Mechanistically, we verified that KLF15 overexpression inhibited ferroptosis by activating the NRF2/GPX4 axis in vitro and in vivo.

Our further mechanistic study verified that KLF15 overexpression increased both cytosolic and nuclear protein levels of NRF2. However, this observation was inconsistent with the report by Gao [26], which suggested that, in SW1353 human chondrocytes, KLF2 stimulates the nuclear translocation of the NRF2 protein, possibly due to the differences among the cell lines and the specificity of the KLF family member. Transcriptional regulatory domains, such as the acidic transactivation domain (TAD), are found in the amino-terminal regions of numerous KLF proteins [46]. Studies have reported that KLF15 can regulate the inflammatory response, and KLF15 is capable of suppressing CCL2 expression in adventitial fibroblasts and negatively regulating the chemokine CXCL1 in cardiac fibroblasts through the TAD [47,48,49]. On the other hand, KLF15 specifically binds to GC-rich sequences in target gene promoters [50]. Our findings showed that KLF15 post-transcriptionally upregulated NRF2 expression.

There have been reports claiming that NRF2 is normally retained in the cytoplasm by binding to the cytoskeleton-associated protein KEAP1 before being degraded by the proteasome. NRF2 levels are low under homeostatic conditions as a result of constant ubiquitination and proteasomal degradation. NRF2 can be removed from the NRF2-KEAP1 dimer and then translocated to the nucleus in response to stimulation [27]. In addition, NRF2 signaling can be activated via the AKT signaling pathway. Glycogen synthase kinase 3 β(GSK3β), which is inhibited by AKT-mediated phosphorylation, mediates NRF2 degradation [27]. There is evidence showing that the histone acetyltransferase p300 promotes NRF2 nuclear localization by enhancing NRF2 protein stability and abundance. Notably, the stabilization of NRF2 was dependent on the acetyltransferase activity of p300 [51,52,53]. This study validated that KLF15 stabilized NRF2 by attenuating its ubiquitin-mediated degradation. However, the exact relationship between KLF15 and NRF2 still remains a mystery and requires further in-depth investigation. One previous study reported that KLF15 interacts directly with the histone acetyltransferase p300 to alter the acetylation status and activity of the proinflammatory factor NF-κB [47]. Wang et al. [54] demonstrated that KLF15 reduces ischemia-induced apoptosis by regulating the p38/MAPK signaling pathway. Additionally, multiple studies recently showed that KLF15 exerts its effects via the AKT-mediated signaling pathway [55,56,57]. As a result, these approaches may be used to further comprehend the involvement of KLF15 and NRF2/GPX4 signaling in AKI. To what extent KLF15 is involved in the regulation of NRF2 needs to be determined by further research.

## 4. Materials and Methods

### 4.1. Animals

Male C57BL/6 mice (10−12 weeks old and weighing 20–25 g) were purchased from WuKong Biotechnology Company (Nanjing, China). KLF15-knockout transgenic mice were purchased and validated by Nanjing GemPharmatech Company. A 12/12 h light/dark cycle and free access to food and water were provided for the animals. All experimental procedures were approved and conducted in accordance with the institutional guidelines for animal care.

Folic acid nephropathy, which is a classic model of kidney tubulointerstitial injury and inflammation, was induced by treatment with FA (i.p., 100 mg·kg^−1^, once daily for 7 consecutive days) in 0.3 mol/L sodium bicarbonate, as reported previously [23]. There were six mice each in the control group and the FA group, which were composed of wild type (WT) or knockout (KO) mice, respectively. The administration of folic acid was performed as described above. Twenty-four hours after the final dose, the mice were euthanized. During the euthanasia process, blood and kidney samples were extracted.

### 4.2. Renal Function, Histology, and Immunohistochemistry

Fresh kidney tissues were acquired, quickly formalin-fixed, and then paraffin-embedded before being cut into 2 μm slices for Periodic Acid-Schiff stain (PAS) staining to determine morphological alterations in the cortex and medulla. Two crucial indicators of kidney function were detected in accordance with the manufacturer’s instructions: serum creatinine (Nanjing Jiancheng Bioengineering Institute, C011-2-1, Nanjing, China) and blood urea nitrogen (Nanjing Jiancheng Bioengineering Institute, A003-2, Nanjing, China). Antigenic recovery for immunohistochemistry was carried out as follows: the slices were placed in 0.01 mol/L pH 6.0 citric acid buffer, heated in a microwave until boiling, and then held at 95 °C or higher for 20 min before cooling naturally to room temperature. Then, primary antibodies against SLC7A11 (Cell Signaling Technology, Danvers, MA, USA), GPX4 (Abcam, Waltham, MA, USA), and 4-hydroxynonenal (Abcam) were added to the slides, diluted with 2% casein in bovine serum albumin to the required concentration, and incubated overnight at 4 °C. The secondary antibodies (HRP secondary antibodies, Cat Nos. KIT-5002 or KIT-5005; MXB Biotechnologies, Fuzhou, China) were added and incubated for one hour at room temperature. To capture images, an Olympus BX41 microscope was used.

### 4.3. Detection of MDA and GSH Levels

The malondialdehyde (MDA) test kit (A003-1, Nanjing Jiancheng Bioengineering Institute, Nanjing, China) and micro reduced glutathione (GSH) assay kit (A006-2-1, Nanjing Jiancheng Bioengineering Institute, Nanjing, China) were used to measure the amounts of malondialdehyde (MDA) and glutathione (GSH) in the tissues and cells.

### 4.4. Cell Culture, Transfection, and Drug Treatments

Immortalized human tubular epithelial cells (HK2 cells) were cultured in DMEM/F12 medium supplemented with 10% fetal bovine serum. Human embryonic kidney (HEK 293) cells were cultured in high-glucose DMEM containing 10% fetal bovine serum and incubated at 37 °C with 5% CO_2_. HK2 cells in six-well plates were prepared for transfection. Then, jetPRIME in vitro DNA & shRNA transfection reagent (PolyPlus Transfection, pt-114-15) was used to transfect plasmids or shRNA, according to the manufacturer’s instructions. In brief, 200 µL of jetPRIME buffer was combined with 2 µg of the plasmid and vortexed. The transfection mixture was administered to the cells in media containing serum after adding 4 µL of jetPRIME reagent and incubated for 10 min. To transfect shRNA, 110 pmole of shRNA was diluted in 200 µL of jetPRIME buffer and vortexed. The transfection mixture was added to the cells in a medium containing serum after adding 3 µL of jetPRIME reagent and incubated for 10 min. The cells were harvested after 48 h of transfection. The cells were treated with 1 µM of erastin (MCE, HY-15763) for 24 h and 10 µM of MG132 (MCE, HY-13259) for 2 h before being harvested.

### 4.5. Cell Viability Assay

Cells were cultured in 96-well plates and treated with drugs at the appropriate times. The CCK-8 Cell Counting Kit was used to assess cell viability (Vazyme, Nanjing, China).

### 4.6. Real-Time Quantitative PCR

Total RNA was extracted from HK2 cells and renal cortex tissues by the FastPure Cell/Tissue Total RNA Isolation Kit (Vazyme, Nanjing, China). In addition, mRNA was reverse-transcribed with an RT2 First Strand Kit (330401, Qiagen, Venlo, Netherlands). The cDNA then served as the template for SYBR real-time PCR. The primer sequences are presented in Table 1. All reactions were run in triplicate on a Real-Time PCR Detection System (Bio-Rad, Hercules, CA, USA).

### 4.7. Western Blot Analysis

Total protein extracts from HK2 cells and mouse renal cortex tissues were prepared as previously described. Equal amounts of proteins were separated by SDS–PAGE and transferred to polyvinylidene fluoride membranes (Millipore, Burlington, MA, USA). After being blocked with 5% skimmed milk for 1 h at room temperature, the membranes were incubated overnight at 4 °C with the following primary antibodies: mouse anti-KLF15 (Santa Cruz, CA, USA), rat anti-KIM-1, rabbit anti-SLC7A11 (Cell Signaling Technology, Danvers, MA, USA), rabbit anti-GPX4 (Abcam, Waltham, MA, USA), rabbit anti-NRF2 (Abcam), rabbit anti-KEAP1 (Proteintech, Rosemont, IL, USA), rabbit anti-Histone3 (H3, Proteintech), and rabbit anti-GAPDH (Proteintech). After being washed, the membranes were incubated with the secondary antibody for one hour. Finally, ECL plus chemiluminescence substrate was used to visualize the protein bands. ImageJ software v1.8.0 was used for relative protein quantification.

### 4.8. Lipid ROS Assay Using Flow Cytometry

Using C11 BODIPY 581/591 (Invitrogen, Carlsbad, CA, USA) as a molecular probe, cellular lipid ROS production was assessed. A 1 mL volume of a medium containing 5 μM C11 BODIPY 581/591 dye was used, and the cells were incubated for an additional 30 min. The cells were washed three times with phosphate-buffered saline (PBS) at the end of the experiment and suspended in 500 µL PBS. Intracellular fluorescence was measured by flow cytometry.

### 4.9. Iron Measurements

Fresh kidney tissues were immediately homogenized with PBS. Following centrifugation, the supernatant was obtained. As directed by the manufacturer, iron levels were measured using an Iron Assay Kit (Abcam).

### 4.10. Co-Immunoprecipitation Assay

For the Co-immunoprecipitation (Co-IP) experiment (Sigma-Aldrich, St. Louis, MO, USA), cells were lysed in RIPA buffer with 10 mmol/L N-ethylmaleimide and a mixture of mammalian protease and phosphatase inhibitors. The filters were then incubated with NRF2 antibody and protein A-agar beads (Cell Signaling Technology, Danvers, MA, USA) at 4 °C for 1 h to produce an immunoprecipitated (IP) sample. The IP sample and 10 g of the input proteins were analyzed by Western blotting using the KLF15 antibody.

### 4.11. Preparation of Nuclear and Cytosolic Fractions

According to the manufacturer’s instructions, the Nuclear and Cytoplasmic Protein Extraction Kit (Inventbiotech, Beijing, China) was used to prepare the nuclear and cytosolic fractions.

### 4.12. Statistical Analysis

All data are displayed as the mean ± standard deviation (SD). SPSS version 23.0 software (SPSS) was used to perform statistical analyses. Student’s *t* test was used for comparisons between the two groups, one-way ANOVA was used when there were more than two groups, and *p* < 0.05 was considered statistically significant.

## 5. Conclusions

In summary, we clearly demonstrated that KLF15 could protect against FA-induced AKI by inhibiting ferroptosis, and this beneficial impact was mediated by the NRF2/GPX4 axis. Taken together, our results provide new insights into the molecular basis of the role of KLF15 in ferroptosis and have implications that KLF15 might be a prospective therapeutic target for preventing the development of AKI.

## Figures and Tables

**Table 1:** The primers for real-time quantitative PCR.

Number	Gene Name	Forward Primer	Reverse Primer
1	h-KLF15	AGGTTCTCGCGCTCTGACG	ACCTTGATGTGCTTGGAGAGG
2	h-NRF2	TCTGGAAAGGACCGTTGTCG	GCCAAGTAGTGTGTCTCCATAG
3	h-GPX4	GAGGCAAGACCGAAGTAAACTAC	CCGAACTGGTTACACGGGAA
4	h-SLC7A11	GGTTGCCCTTTCCCTCTATTC	CCTGGGTTTCTTGTCCCATATAA
5	h-HO-1	GGAAATCATCCCTTGCACGC	TGTTTGAACTTGGTGGGGCT
6	h-NQO1	CATTGCAGTGGTTTGGGGTG	TCTGGAAAGGACCGTTGTCG
7	h-GCLM	GAGTTGCACAGCTGGATTCT	CCTCCCAGTAAGGCTGTAAATG
8	m-KLF15	CCCAGCTTCTAGTCAACATCC	GCGCAATTCGCACAAACT
9	m-KIM-1	CCAGGCGCTGTGGATTCTTA	TGTACCGACTGCTCTTCTGATAGG
10	m-NGAL	TGGCCCTGAGTGTCATGTG	CTCTTGTAGCTCATAGATGGTGC
11	m-NRF2	CAATGAGGTTTCTTCGGCTACG	AAGACTGGGCTCTCGATGTG

KLF15, Krüppel-like factor 15; NRF2, nuclear factor erythroid 2-related factor 2; GPX4, glutathione peroxidase 4; HO-1, heme oxygenase-1; NQO1, NADPH quinone oxidoreductase 1; GCLM, glutamate-cysteine ligase modifier subunit; KIM-1, kidney injury molecule 1; NGAL, neutrophil gelatinase-associated lipocalin.

## Data Availability

Data available on request due to restrictions, e.g., privacy or ethical.

## Supplementary Materials

The supporting information can be downloaded at: https://www.mdpi.com/article/10.3390/ijms241914530/s1.
