# Supplementary material for: Krüppel-like Factor 15 Suppresses Ferroptosis by Activating an NRF2/GPX4 Signal to Protect against Folic Acid-Induced Acute Kidney Injury
Source: Int J Mol Sci. 2023 Sep 26;24(19):14530. doi: 10.3390/ijms241914530 (PMC10572468; doi:10.3390/ijms241914530)
Supplement: Supplementary file 1 [file ijms-24-14530-s001.zip › ijms-2575469-supplementary.pdf]

Fig. S1. KEAP1 protein level was evaluated by Western blot analysis in KLF15 knockdown or overexpression HK2 cells. A-D, Protein levels of KEAP1 were detected by western blot. KEAP1, Kelch-like ECH-associated protein 1; KLF15, Krüppel-like factor 15; HK2, human tubular epithelial cells; ns, none sense.

Fig. S2. Co-IP assay did not show the interaction between KLF15 and NRF2 in HK2 and HEK 293 cells. KLF15, Krüppel-like factor 15; NRF2, nuclear factor erythroid 2-related factor 2; HK2, human tubular epithelial cells.

S1

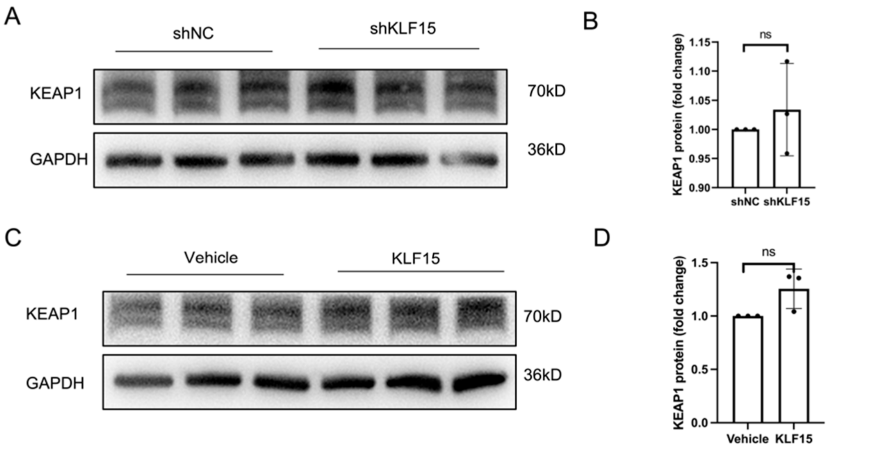
A
shNC
shKLF15
KEAP1
70kD
GAPDH
36kD
B
ns
KEAP1 protein (fold change)
1.15
1.10
1.05
1.00
0.95
0.90
shNC
shKLF15
C
Vehicle
KLF15
KEAP1
70kD
GAPDH
36kD
D
ns
KEAP1 protein (fold change)
1.5
1.0
0.5
0.0
Vehicle
KLF15

S2

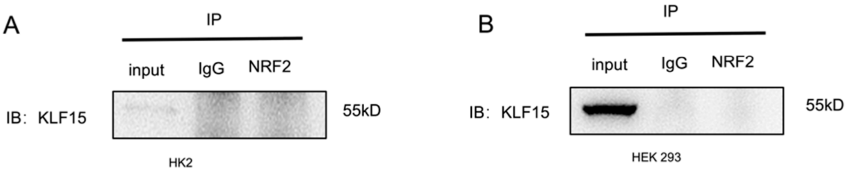
A
IP
input
IgG
NRF2
IB：KLF15
55kD
HK2
B
IP
input
IgG
NRF2
IB：KLF15
55kD
HEK 293
